# Optimizing IMPULSED Acquisition Protocols for Clinical 3T Scanners Through Bayesian Experimental Design

**DOI:** 10.1002/mrm.70579

**Published:** 2026-09-14

**Authors:** Yan Dai, Xun Jia, Todd Aguilera, Kai Jiang, Arely Perez Rodriguez, Isabelle Vanhaezebrouck, Jie Deng

**Affiliations:** ^1^ Department of Radiation Oncology University of Texas Southwestern Medical Center Dallas Texas USA; ^2^ Department of Radiation Oncology and Molecular Radiation Sciences Johns Hopkins University Baltimore Maryland USA

**Keywords:** experimental design, Imaging Microstructural Parameters Using Limited Spectrally Edited Diffusion model, model fitting, quantitative MRI, robustness

## Abstract

**Purpose:**

To optimize diffusion MRI acquisition protocols for Imaging Microstructural Parameters Using Limited Spectrally Edited Diffusion (IMPULSED) model at clinical 3T scanner using Bayesian experimental design, enabling accurate cellular‐scale parameter estimation under realistic scan time and scanner hardware constraints.

**Methods:**

Expected Information Gain (EIG) was used as the optimization objective to maximize the information content of acquired measurements for IMPULSED model fitting. Bayesian optimization with Gaussian process surrogates efficiently searched the high‐dimensional acquisition parameter space, including pulse types (PGSE, OGSEn1, and OGSEn2), diffusion times, and *b* values. Optimized protocols were systematically evaluated against a heuristically designed baseline protocol through simulation studies assessing classification accuracy and parameter estimation performance across SNR levels of 5–40. Robustness to optimization assumptions was examined by varying prior distributions and assumed SNR. In vivo validation was performed using canine tumor data acquired at 3T.

**Results:**

The optimized protocol eliminated OGSEn2 acquisitions, concentrated measurements at high *b* values (maximum 2000 s/mm^2^ for PGSE, 1220 s/mm^2^ for OGSEn1), employing concurrently optimized diffusion timing. Compared to the baseline protocol, the optimized design achieved superior classification accuracy for distinguishing cell populations and reduced parameter estimation error across biologically relevant parameter ranges at various SNRs. Performance advantages were consistent across diverse optimization scenarios, demonstrating robustness to prior knowledge and noise assumptions. In vivo parameter maps showed substantially improved quality and smoothness.

**Conclusion:**

Bayesian optimization substantially improves IMPULSED acquisition design for clinical 3T scanners. This principled, algorithm‐agnostic framework enables accurate diffusion MRI cytometry under clinical constraints, with potential applications to tumor characterization and treatment monitoring.

## 1 Introduction

Diffusion magnetic resonance imaging (dMRI) characterizes tissue microstructure by measuring signal attenuation caused by water molecule displacement under diffusion‐weighted gradients [1, 2]. Quantitative parameters are obtained by fitting biophysical models to the acquired signals, providing imaging biomarkers about cell microenvironment [3, 4, 5, 6]. The accuracy and robustness of these biomarkers depend on both measurement quality, determined by acquisition strategy and signal‐to‐noise ratio (SNR), and the geometry of the inverse problem underlying parameter fitting [7, 8]. While considerable effort has focused on improving parameter estimation algorithms [9, 10, 11, 12, 13, 14], the design of the acquisition protocol itself remains a critical yet often under‐optimized factor. Most clinical dMRI protocols are still designed heuristically rather than through principled experimental design [15, 16, 17, 18], which can limit parameter identifiability for complex diffusion models.

Experimental design optimization generally involves defining a target function and searching for acquisition parameters that maximize this objective. Within dMRI, experimental design optimization has been investigated for several commonly used models such as the apparent diffusion coefficient (ADC) and intravoxel incoherent motion (IVIM) models. Existing methods typically optimize the selection of *b* values under scan‐time constraints using target functions include variance‐based metrics such as parameter estimation error [19, 20, 21, 22, 23, 24, 25], fisher information matrix [26], or Cramér–Rao lower bound (CRLB) [27, 28, 29], and task‐based objectives such as maximizing class distinguishability [30, 31] or preserving linearity between estimations and ground truth values [32, 33, 34]. Because analytical gradients with respect to acquisition parameters are often unavailable or computationally intractable, these objectives are typically treated as black boxes and optimized using numerical strategies such as brute‐force search [21, 22, 23, 25, 29, 30, 32], genetic algorithms [19, 20, 24], or surrogate modeling approaches [35, 36].

Critically, these strategies rely on specific parameter estimation algorithms, limiting generalizability, and approximate uncertainty using simplified lower‐order metrics, which may be inadequate for complex qMRI models. Consequently, they are not directly extensible to modern diffusion models with high‐dimensional parameter spaces and strongly ill‐conditioned inverse mappings, such as the Imaging Microstructural Parameters Using Limited Spectrally Edited Diffusion (IMPULSED) model [37] considered in this work.

IMPULSED is an advanced dMRI framework modeling water displacement in intracellular and extracellular compartments across multiple effective diffusion times with a combination of pulsed gradient spin echo (PGSE) and oscillating gradient spin echo (OGSE) waveforms [38, 39]. It enables estimation of cellular‐scale parameters such as cell size and intracellular volume fraction [37], which have shown specificity for characterizing radiation‐induced biological changes in both preclinical and clinical studies [40, 41, 42]. Although successful implementations have been demonstrated on higher‐field scanners (e.g., 4.7T, 7T) using empirically selected diffusion times and evenly distributed *b* values [17, 37, 43], translating IMPULSED model to clinical 3T systems remains challenging. Reduced gradient strength limits the highest achievable diffusion coefficient, and clinically feasible scan times restrict compensating for low SNR through signal averaging. In addition, the highly nonlinear nature of IMPULSED model leads to an ill‐conditioned parameter estimation problem. These challenges necessitate optimized acquisition designs that maximize the information content of the measurements. However, experimental design for IMPULSED model fitting involves a high‐dimensional parameter space and an extremely ill‐conditioned parameter‐fitting landscape, making many existing dMRI experimental design optimization approaches inapplicable.

In this work, we formulate IMPULSED protocol optimization as an information‐theory‐based problem, using Expected Information Gain (EIG) as the target function [44, 45]. To our knowledge, this is the first study to apply information‐theoretic experimental design to dMRI acquisition. Bayesian optimization with Gaussian process (GP) surrogate modeling is employed to efficiently search the high‐dimensional acquisition parameter space. This framework directly maximizes the information content acquired and is independent of downstream parameter estimation algorithms, enabling general application across different fitting strategies. We systematically analyzed the effectiveness and robustness of the proposed experimental design optimization framework for the IMPULSED model under realistic clinical constraints.

## 2 Methods

### 2.1 IMPULSED Model

IMPULSED model is a two‐compartment framework assuming isotropic water diffusion both inside and outside sphere‐shaped cells with impermeable membranes. Conceptually, it connects the observed diffusion signal S with contributions from restricted intracellular and extracellular diffusion. The signal is expressed as a weighted sum of intracellular and extracellular contributions: $S=V_{\text{in}}\cdot S_{\text{in}}+\left(1-V_{\text{in}}\right)\cdot S_{\mathrm{ex}}$, where $V_{\text{in}}$ is the intracellular volume fraction, and $S_{\text{in}}$ and $S_{\mathrm{ex}}$ are signals from the intra‐ and extracellular compartments, respectively. The IMPULSED model describes diffusion signal decay arising from PGSE, OGSEn1, and OGSEn2 acquisition pulses. $S_{\text{in}}$ is expressed as Equation (1) for PGSE and Equation (2) for OGSEs.

(1) $$\begin{array}{cc} S_{\text{in}}^{\text{PGSE}} & =\exp\left(-2{\left(\frac{\gamma G}{D_{\text{in}}}\right)}^{2}\sum_{n}\frac{B_{n}}{\lambda_{n}^{2}}\left\{\lambda_{n}D_{\text{in}}\delta -1+\exp\left(-\lambda_{n}D_{\text{in}}\delta \right)\right.\right.\\ & \;\left.\left.+\exp\left(-\lambda_{n}D_{\text{in}}\Delta \right)\left(1-\cosh\left(\lambda_{n}D_{\text{in}}\delta \right)\right)\right\}\right), \end{array}$$

(2) $$\begin{array}{cc} S_{\text{in}}^{\text{OGSE}} & =\exp\left(-2{\left(\gamma G\right)}^{2}\sum_{n}\frac{B_{n}\lambda_{n}^{2}D_{\text{in}}^{2}}{{\left(\lambda_{n}^{2}D_{\text{in}}^{2}+4\pi^{2}f^{2}\right)}^{2}}\left\{\frac{\left(\lambda_{n}^{2}D_{\text{in}}^{2}+4\pi^{2}f^{2}\right)}{\lambda_{n}D_{\text{in}}}\left[\frac{\delta }{2}+\frac{\sin\left(4\pi f\delta \right)}{8\pi f}\right]\right.\right.\\ & \;-\left.\left.1+\exp\left(-\lambda_{n}D_{\text{in}}\delta \right)+\exp\left(-\lambda_{n}D_{\text{in}}\Delta \right)\left(1-\cosh\left(\lambda_{n}D_{\text{in}}\delta \right)\right)\right\}\right). \end{array}$$

Microstructural parameters, including cell radius (R), intracellular volume fraction ($V_{\text{in}}$), intracellular diffusion coefficient ($D_{\text{in}}$), and extracellular diffusion parameters ($D_{\mathrm{ex}}$, $\beta_{\mathrm{ex}}$) can be estimated by fitting the IMPULSED model. Here, γ denotes the gyromagnetic ratio; δ and Δ represent the duration of diffusion encoding gradient pulse and the time interval between the two diffusion encoding gradient pulses around the 180° refocusing pulse, respectively; and G denotes the diffusion encoding gradient amplitude. $\lambda_{n}$ and $B_{n}$ are structure‐dependent parameters determined by R. The oscillating frequency is defined as f=n/δ, with n=0,1,2 for PGSE, OGSEn1 and OGSEn2, respectively. Then $S_{\mathrm{ex}}$ is expressed as $S_{\mathrm{ex}}=\exp\left(-b\left(D_{\mathrm{ex}}+\beta_{\mathrm{ex}}\cdot f\right)\right)$. This model enables measurement of diffusion behavior across a wide range of $t_{\text{diff}}$ through the combined use of PGSE, OGSEn1, and OGSEn2 waveforms. For PGSE, the effective diffusion time is given by $t_{\text{diff}}=\Delta -\delta /3$, whereas for OGSE, $t_{\text{diff}}=\delta /4n$. The b‐value commonly used in dMRI does not explicitly appear in this representation, but is instead decomposed into g,δ, and Δ, such that $b=\gamma^{2}\int_{0}^{+\infty }\mathrm{dt}{\left|\int_{0}^{t}{\mathrm{dt}}^{\prime }g\left(t^{\prime }\right)\right|}^{2}$, where g(t) denotes the diffusion encoding gradient waveform with amplitude G. Thus, given fixed δ and Δ, the b‐value can be considered as a one‐to‐one mapping of G. The pulse sequence parameters are illustrated in Figure S1.

### 2.2 Tunable Protocol Parameters for Experimental Design Optimization

The tunable parameters for each acquisition include pulse type (PGSE, OGSEn1, or OGSEn2) and the corresponding timing parameters (δ and Δ), which determine the effective diffusion time $t_{\text{diff}}$. In addition, the b‐values, or equivalently, the diffusion encoding gradient amplitude G for a given pulse type, need to be determined. To keep relaxation‐related effects constant, the echo time (TE) and repetition time (TR) were kept the same for all pulses (*n* = 0, 1, 2).

In our implementation on a clinical scanner (3T Philips Achieva MR system), we adopted timing parameters following the baseline protocol applied by Xu et al. [17], with a gradient pulse duration of 41 ms, a pulse gap of 10 ms between gradient pulses, yielding the same TE = 103 ms. TR was matched to their reported value (4500 ms). The maximum gradient amplitude G was set to 80 mT/m, according to the hardware constraints of the 3T system.

The bounds for timing parameters were determined to ensure physically realizable protocols. First, the lower bound of δ for each pulse type was constrained to ensure the maximum achievable *b*‐value exceeded 100 s/mm^2^: $\delta_{\text{PGSE}}$ ≥ 10 ms, $\delta_{\text{OGSEn}1}$ ≥ 19 ms, and $\delta_{\text{OGSEn}2}$ ≥ 30 ms. Given these minimum δ values, the upper bound of Δ for each sequence type was derived as: $\Delta_{\max}$ = 2×pulse duration − $\text{pulse}\;\mathrm{gap}-\delta_{\min}$, yielding $\Delta_{\text{PGSE}}$ ≤ 82 ms, $\Delta_{\text{OGSEn}1}$ ≤ 73 ms, and $\Delta_{\text{OGSEn}2}$ ≤ 62 ms. The lower bound of Δ was set to ($\delta_{\min}$ + pulse gap), accounting for the duration of the 180° refocusing pulse. For each specific Δ during optimization, the upper bound of δ was dynamically determined as min(Δ−PulseGap,2×pulse duration − pulsegap−Δ).

To avoid non‐Gaussian diffusion effects associated with excessively high b‐values and considering the highest b‐value 1800 s/mm^2^ applied by Xu et al., the b‐value was capped at 2000 s/mm^2^. Following the acquisition design of Xu et al. [17], a complete IMPULSED acquisition protocol was limited to 13 nonzero *b*‐value measurements plus 3 zero‐b measurements for each pulse type, corresponding to a total scan time of approximately 3.5 min on our 3T scanner. This consistent protocol structure enables direct comparison between our optimized acquisition schemes and the baseline protocol.

### 2.3 Target Function: EIG

EIG, derived from Bayesian experimental design theory [44], was used as the target function to quantify the amount of information obtained about the model parameters θ from measured dMRI signals $S_{m}$ acquired under experimental design d. EIG is defined in Equation (3).

(3) $$\begin{array}{cc} \mathrm{EIG}\left(d\right) & =E_{P\left(S_{m}|d\right)}\left[E_{P\left(\theta |S_{m};d\right)}\left[\ln P\left(\theta |S_{m};d\right)\right]-E_{P\left(\theta \right)}\left[\ln P\left(\theta \right)\right]\right]\\ & =E_{P\left(\theta \right)P\left(S_{m}|\theta ;d\right)}\left[\ln P\left(S_{m}|\theta ;d\right)-\ln P\left(S_{m}|d\right)\right], \end{array}$$

where $S_{m}=S_{m,1},S_{m,2},\ldots ,S_{m,K}$ represents the measured dMRI signals for a voxel with microstructural parameters $\theta =\left(R,V_{\text{in}},D_{\text{in}},D_{\mathrm{ex}},\beta_{\mathrm{ex}}\right)$ acquired using experimental design d. The subscript m labels “measured,” distinguishing the noisy measurement from the theoretical noiseless signal S defined in Section 2.1. The semicolon “;d” indicates that d is not a conditioning random variable, since the parameters θ are independent of the experimental design d. We retain “;d” throughout to emphasize the impact of d in the qMRI process. EIG measures the expected reduction in entropy from the prior to the posterior distributions of the model parameters, as is shown in the first row of Equation (3). It is also the information gained by performing the measurement with experimental design d and, equivalently, the mutual information between θ and $S_{m}$ conditional on d. Given the Bayes' rule, $P\left(\theta |S_{m};d\right)=P\left(\theta |S_{m}\right)=P\left(S_{m}|\theta \right)P\left(\theta \right)/P\left(S_{m}\right)$, the first row yields the second row of Equation (3), which is used in our numerical estimation.

EIG was evaluated numerically as follows. The prior distribution P(θ) was assumed to be uniform within physiologically‐relevant ranges [37]: $R\in \left[2,12\right]\;\mathrm{\mu m},D_{\text{in}}\in \left[0.2,2\right]\;\mu m^{2}/\mathrm{ms},V_{\text{in}}\in \left[0.2,0.8\right],D_{\mathrm{ex}}\in \left[0.2,2\right]\;\mu m^{2}/\mathrm{ms}$ and $\beta_{\mathrm{ex}}\in \left[0,6\right]\;\mu m^{2}$. To approximate the expectation over the prior, a total of 6×4×6×4×4=2304 parameter samples were generated using grid‐based sampling within this space, with denser sampling applied for cell radius R and intracellular volume fraction $V_{\text{in}}$. Assuming additive Gaussian noise in the measured signals, which provides a good approximation to Rician noise at SNR>3, then $P\left(S_{m}|\theta ;d\right)=\prod_{i=1}^{K}N\;\left(S_{m,i};\text{IMPULSED}\left(\theta ,d\right),\sigma_{i}\right)$, where N(.) represents a Gaussian distribution with mean ${\text{IMPULSED}}_{i}\left(\theta ,d\right)$ and noise level $\sigma_{i}$. Here, ${\text{IMPULSED}}_{i}\left(\theta ,d\right)$ represents the noiseless IMPULSED signal for the ith acquisition setting under design d. The expectation over measurement noise was estimated using Monte Carlo sampling. For each parameter sample, 100 noisy signal realizations $P\left(S_{m}|\theta ;d\right)$ were generated by repeatedly adding Gaussian noise to the noiseless signal at an SNR=20, representative of typical clinical imaging conditions. The marginal $P\left(S_{m};d\right)=\int P\left(S_{m}|\theta ;d\right)\;P\left(\theta \right)\;d\theta$ was estimated by averaging the likelihood over the same 2304‐point prior grid, that is, $P\left(S_{m};d\right)\approx \frac{1}{2304}\Sigma_{k}\;P\left(S_{m}|\theta_{k};d\right)$. The EIG was then computed by averaging over all simulated measurements and parameter samples. By maximizing this target function, the optimal acquisition design $d^{*}$ was identified.

### 2.4 Bayesian Optimization Procedure

The acquisition protocol design was formulated as a 32‐dimensional continuous optimization problem as Bayesian Optimization input. Specifically, a protocol consisting of 13 individual measurements was encoded as a vector with 6+13×2 elements. The six shared parameters correspond to the timing variables δ and Δ for each of the three diffusion encoding gradients waveform types (PGSE, OGSEn1, or OGSEn2). Each of the 13 acquisitions contributes 2 parameters: the diffusion encoding gradient pulse type indicator that specifies whether the acquisition uses PGSE, OGSEn1, or OGSEn2, and its corresponding *b*‐value. To enable optimization within a continuous parameter space, the gradient pulse type indicator was represented by a continuous indicator variable in the interval [0,1]. During protocol evaluation, this variable was mapped to the three discrete waveform types through interval partitioning: values in [0, 1/3) correspond to PGSE, values in [1/3, 2/3) correspond to OGSEn1, and values in [2/3, 1] correspond to OGSEn2. Each dimension of the vector was normalized from its physical bounds to the unit interval [0,1], enabling efficient exploration of the parameter space by the GP surrogate model.

Gaussian process‐based [46] Bayesian optimization was employed to efficiently explore the EIG as a function of acquisition parameters. A Matérn kernel was selected to model the structure of the EIG surface, enabling a flexible surrogate representation of the underlying black‐box objective. The optimization process was initiated with 300 protocol evaluations generated via quasi‐random Sobol sampling, followed by 500 sequential optimization iterations. At each iteration, new candidate acquisition designs were selected by maximizing the log expected improvement acquisition function, balancing exploration and exploitation. The Bayesian optimization was implemented using BoTorch [47].

### 2.5 Evaluation

Experimental designs were evaluated by comparing an EIG‐optimized protocol under uniform priors at SNR 20 (*Optimized SNR 20*) with a literature‐based [17] baseline protocol (*Baseline*). Simulations assessed classification and parameter estimation performance under different noise levels, as well as the effects of optimization assumptions such as SNR and prior distributions on protocol structure. The two protocols were also qualitatively compared in a canine scan study.

#### 2.5.1 Simulation Study

##### 2.5.1.1 Classification

###### 2.5.1.1.1 Data Generation

Synthetic datasets were generated to evaluate the classification performance of different IMPULSED acquisition protocols. Two biologically relevant microstructural variations were simulated: immune‐cell infiltration, affecting cell radius R, and tumor apoptosis, affecting intracellular volume fraction $V_{\text{in}}$. For each experiment, only one parameter (R or $V_{\text{in}}$) was varied across the same ground truth values used for prior sampling, yielding six distinct “tissue states.” All other parameters were fixed at physiologically representative values [37] ($R=7\mathrm{\mu m},V_{\text{in}}=0.6,D_{\mathrm{ex}}=1.1\;\mu m^{2}/\mathrm{ms}$, $D_{\text{in}}=0.9\;\mu m^{2}/\mathrm{ms}$ and $\beta_{\mathrm{ex}}=2\;\mu m^{2}$). For each parameter setting, 500 independent noisy signal realizations were generated.

Noisy signals were simulated using a Rician noise model. Noise‐free signals were first generated from the IMPULSED model for each acquisition protocol, after which complex Gaussian noise with standard deviation σ was added to the real and imaginary components, followed by magnitude reconstruction. Four noise levels corresponding to SNR of 5, 10, 20, and 40 were evaluated, where SNR=1/σ.

An additional classification study modeling T‐cell infiltration into tumor tissue was performed to evaluate protocol performance under biologically realistic heterogeneity (Supplementary S1).

###### 2.5.1.1.2 Classification Algorithm

Classification was performed using a Random Forest classifier. Each noisy signal vector, consisting of signal intensities from all acquisitions using a given protocol, served as the input feature. Integeral Class labels (0–5) were assigned, corresponding to increasing ground truth values of R or $V_{\text{in}}$.

###### 2.5.1.1.3 Evaluation Metric

Classification performance was evaluated using 10‐fold cross‐validation. For each protocol and SNR level, the mean classification accuracy and standard deviation across folds were computed. Principal component analysis (PCA) was additionally applied to visualize class separability in the signal space.

##### 2.5.1.2 Quantification

###### 2.5.1.2.1 Data Generation

A synthetic dataset containing 100 000 parameter combinations was generated by uniformly sampling microstructural parameters within predefined physiologically relevant ranges. For each parameter set, noisy dMRI signals were simulated using a Rician noise model at SNRs of 5, 10, 20, and 40, following the procedure described above. All signals were normalized using b0 acquisitions prior to parameter fitting.

###### 2.5.1.2.2 Quantification Algorithm

Parameter estimation was performed using nonlinear least‐squares (NLLS) optimization implemented with the Trust Region Reflective (TRF) algorithm. To improve fitting stability, $D_{\text{in}}$ was fixed at 1.58 $\mu m^{2}/\mathrm{ms}$ and $\beta_{\mathrm{ex}}$ at 2 $\mu m^{2}$, leaving three free parameters (R, $V_{\text{in}}$ and $D_{\mathrm{ex}}$) [17]. Parameter bounds were set to $R\in \left[0.2,15\right]\;\mathrm{\mu m},V_{\text{in}}\in \left[0,1.0\right],$ and $D_{\mathrm{ex}}\in \left[0,3\right]\;\mu m^{2}/\mathrm{ms}$. Initial parameter values were randomly sampled within the predefined physiologically‐relevant ranges for each optimization run.

###### 2.5.1.2.3 Evaluation Metric

Quantification accuracy was evaluated using root mean square error (RMSE), normalized by the corresponding upper bounds of the fitting constraints ($R:15\;\mathrm{\mu m},V_{\text{in}}:1.0,D_{\mathrm{ex}}:3\;\mu m^{2}/\mathrm{ms}$). To examine spatial variation in estimation accuracy, the (R,$V_{\text{in}})$ parameter space was discretized into a 5 × 5 grid using bin edges R=[2.0,4.0,6.0,8.0,10.0,12.0]μm and $V_{\text{in}}=\left[0.2,0.32,0.44,0.56,0.68,0.8\right]$, resulting in 25 distinct biophysical regimes spanning from small cells with low intracellular volume to large cells with high intracellular volume. Parameter‐specific RMSE values were computed independently within each bin.

##### 2.5.1.3 Ablation Analysis

Two additional experiments were conducted to isolate the contributions of specific experimental design elements. First, an optimized protocol derived by maximizing EIG under the same uniform prior distribution but assuming a higher SNR of 40 (*Optimized SNR 40*) was evaluated to assess the sensitivity of the optimization outcome to noise assumptions. Second, a modified baseline protocol (*Baseline2*) was constructed by adopting the optimized $t_{\text{diff}}$ from the *Optimized SNR 20* protocol while retaining the *Baseline* b‐value distribution.

##### 2.5.1.4 Dependence of Optimized Protocols on Prior Distributions and SNR

To examine how optimization outcomes depend on prior assumptions and noise conditions, acquisition protocols were optimized under 13 experimental configurations comprising nine prior distributions and four SNR levels.

The prior distributions represented varying degrees of biological knowledge about underlying cell populations. Three configurations for cell radius R (uniform (UR), small‐cell‐biased (SR), and large‐cell‐biased (LR)) were combined with three configurations for intracellular volume fraction $V_{\text{in}}$ (uniform (UVin), small‐$V_{\text{in}}$‐biased (SVin), and large‐$V_{\text{in}}$‐biased (LVin)), this yields eight non‐uniform prior combinations UR‐SVin, UR‐LVin, SR‐UVin, SR‐SVin, SR‐LVin, LR‐UVin, LR‐SVin, and LR‐LVin, aside from UR‐UVin. Each prior distribution was modeled using a linear probability density P(x)∝(1−ratio)·u+ratio, where $u=\left(x-x_{\min}\right)/\left(x_{\max}-x_{\min}\right)$. Ratio of 10 and 0.1 were used to generate the small‐biased and large‐biased priors, respectively. Details of prior distributions are shown in Figure S2. For prior comparisons, the SNR was fixed at 20. Further, to assess SNR assumption dependence, additional optimizations were performed at SNR levels of 5, 10, and 40 using the uniform prior (UR‐UVin).

#### 2.5.2 In‐Vivo Animal Patient Study

To evaluate the optimized experimental design in vivo, dMRI data were acquired from a canine tumor patient on a clinical 3T MRI scanner (Philips Achieva). The patient had a squamous cell carcinoma located in the temporal region dorsal to the left eye and is scheduled to receive 5 fractions of radiation treatment, with a total prescribed dose of 40 Gy delivered at one‐month intervals between fractions. Both the *Baseline* and *Optimized SNR 20* protocols were applied at each acquisition time point. Additional imaging parameters other than diffusion encoding related parameters were matched across both protocols: echo time (TE) = 105 ms, repetition time (TR) = 4500 ms, field of view (FOV) = 192 × 192 mm^2^, voxel size = 2 × 2 × 5 mm^3^, SENSE parallel imaging with acceleration factor of 3, and fat suppression enabled. Total acquisition time was approximately 3.5 min per protocol. A tumor region of interest (ROI) was manually segmented, and the same NLLS algorithm was used to estimate the parameters R, $V_{\text{in}}$, and $D_{\mathrm{ex}}$ for each protocol. Parameter maps were generated for qualitative comparison of estimation stability. The study has been approved by our IACUC committee (protocol number: 2025‐103848‐VCT).

## 3 Results

### 3.1 Experimental Optimization Results

Table 1 summarizes the optimized protocol under uniform priors at SNR 20 (*Optimized SNR 20*) and the Baseline protocol (*Baseline*). The *Optimized SNR 20* protocol exhibits several distinctive features compared to the *Baseline*. Most notably, the optimization eliminated the OGSEn2 pulse, reallocating those measurements to PGSE and OGSEn1 sequences. For OGSEn1, the optimized protocol concentrated at the maximum achievable *b*‐value of 1220 s/mm^2^, utilizing the maximum gradient duration (*δ* = 41 ms, Δ = 51 ms) identical to the *Baseline*, thereby maximizing its effective diffusion time ($t_{\text{diff}}$ = 10.25 ms).

**TABLE 1:** Comparison of optimized and baseline pulse sequence parameters.

Protocol type	Sequence	δ/Δ(ms)	$t_{\text{diff}}\;\left(\mathrm{ms}\right)$	Nonzero b‐value $\left(s/mm^{2}\right)$
Optimized SNR 20	PGSE	33/59	48	688(1), 800(1), 829(1), 931(1), 2000(4)
	OGSEn1	41/51	10.25	1220(5)
Baseline	PGSE	12/74	70	250(1), 500(1), 750(1), 1000(1), 1400(1), 1800(1)
	OGSEn1	41/51	10.25	250(1), 500(1), 750(1), 1000(1)
	OGSEn2	41/51	5.125	100(1), 200(1), 300(1)
Baseline2	PGSE	33/59	48	250(1), 500(1), 750(1), 1000(1), 1400(1),1800(1)
	OGSEn1	41/51	10.25	250(1), 500(1), 750(1), 1000(1)
	OGSEn2	41/51	5.125	100(1), 200(1), 300(1)
Optimized SNR 40	PGSE	26/62	53.33	583(1), 710(1), 767(1), 871(1), 2000(3)
	OGSEn1	41/51	10.25	1220(4)
	OGSEn2	41/51	5.125	298(2)

*Note:* Optimized SNR 20 and Optimized SNR 40 protocol were obtained via Bayesian optimization maximizing Expected Information Gain under UR‐UVin prior distribution with SNR = 20 and SNR = 40 noise assumptions respectively. The protocol applied by Xu et al. [17] was used as Baseline protocol. While Baseline2 protocol was constructed based on Baseline by replacing the timing parameters with that of protocol Optimized SNR 20. All protocols contain 13 nonzero acquisitions. The maximum OGSEn2 *b*‐value (298 s/mm^2^) differs slightly from the baseline protocol (300 s/mm^2^) due to floating‐point precision and minor variations in achievable gradient strength. Numbers in parentheses indicate the number of acquisitions at the corresponding *b*‐value.

Abbreviations: δ, gradient pulse duration; Δ, time interval between two diffusion pulses; $t_{\text{diff}}$, effective diffusion time; OGSE, oscillating gradient spin echo with oscillation number *n*; PGSE, pulsed gradient spin echo.

The PGSE component showed a similar sampling strategy. The optimized protocol allocated eight measurements to PGSE, with sparse sampling at intermediate *b* values (500–1000 s/mm^2^) and dense sampling at the maximum *b*‐value of 2000 s/mm^2^. However, unlike OGSEn1, the PGSE timing parameters (δ = 33 ms, Δ = 59 ms) resulted in a relatively shorter effective diffusion time ($t_{\text{diff}}$ = 48 ms) compared to that for *Baseline* ($t_{\text{diff}}$ = 70 ms). Detailed convergence analysis confirmed successful optimization despite Monte Carlo stochasticity, with clustered *b* values representing near‐equivalent protocols within flat EIG landscape regions (Supplementary S2, Figure S3).

### 3.2 Simulation Study: Superior Performance of Optimized Protocols

#### 3.2.1 Classification Performance

Figure 1 shows classification accuracy for R and $V_{\text{in}}$ across SNR 5‐40. Optimized SNR 20 consistently outperformed Baseline, with greatest improvements at intermediate SNR. PCA of signal vectors with varying R and $V_{\text{in}}$ illustrated enhanced class separability, with the Optimized SNR 20 protocol yielding more distinct clusters than the Baseline protocol for both parameters. The numerical results are shown in Table 2.

**FIGURE 1:**
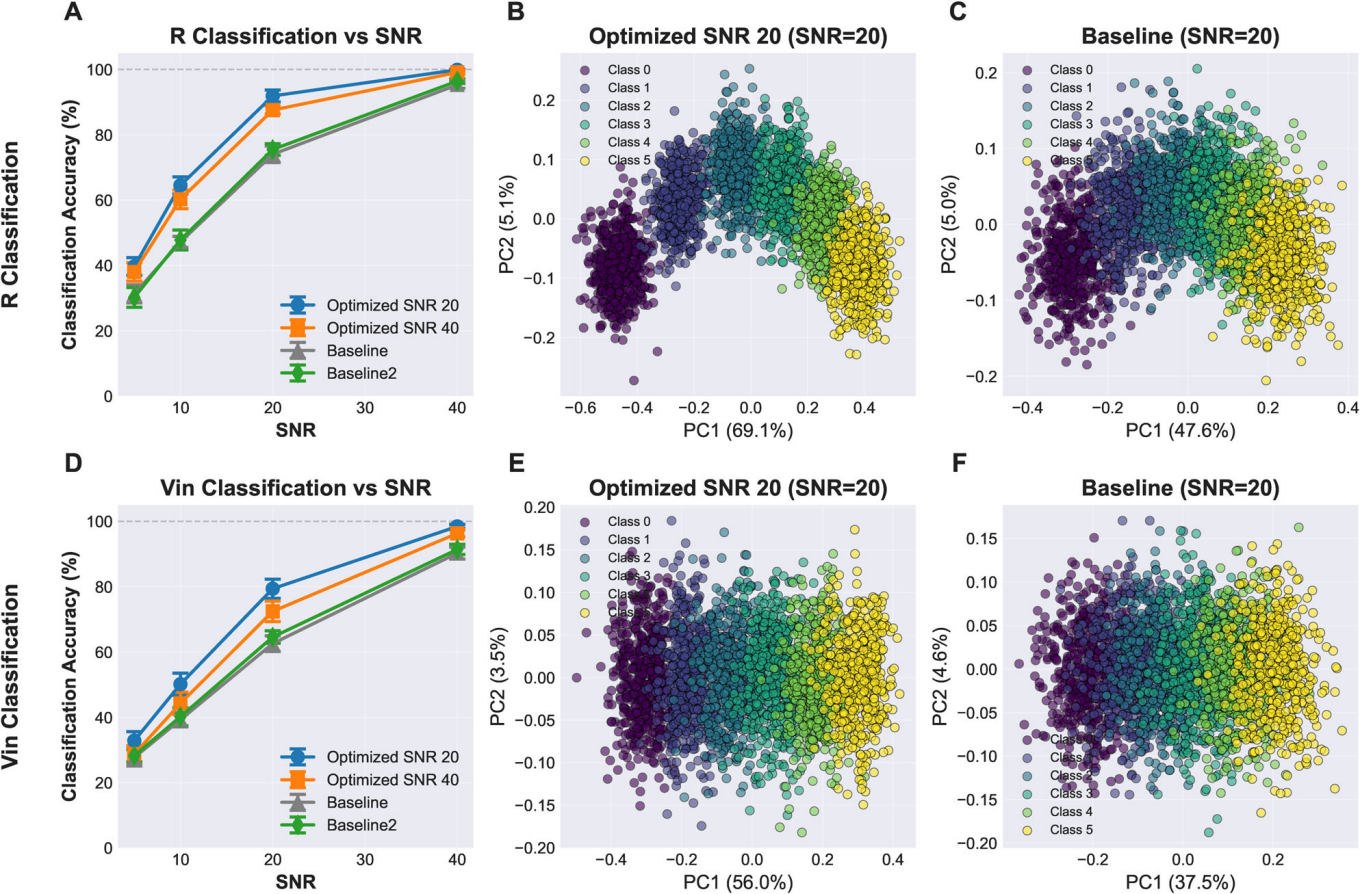
Optimized protocols improve cell classification across noise conditions. Random Forest classification accuracy at SNR levels 5, 10, 20, and 40 for four protocols: *Optimized SNR 20* (blue), *Optimized SNR 40* (orange), *Baseline2* (green), and *Baseline* (gray). Optimized protocols achieve higher accuracy across all noise conditions, with greatest improvements at intermediate SNR. PCA of signal vectors with varying *R* and $V_{\text{in}}$ illustrated enhanced class separability. The *Optimized SNR 20* protocol produced substantially improved separation between classes compared with the *Baseline* protocol for both *R* and $V_{\text{in}}$, yielding distinct and well‐separated clusters in the dominant principal component space.

**TABLE 2:** Random Forest classification accuracy for cell population discrimination.

R classification accuracy (%)				
Protocol	SNR = 5	SNR = 10	SNR = 20	SNR = 40
Optimized SNR 20	39.70 ± 2.73	64.53 ± 2.59	91.90 ± 1.82	99.90 ± 0.15
Optimized SNR 40	38.03 ± 2.87	60.27 ± 2.88	87.57 ± 1.20	99.10 ± 0.54
Baseline	31.30 ± 2.73	47.20 ± 1.69	74.03 ± 2.53	95.30 ± 1.06
Baseline2	30.20 ± 3.02	47.80 ± 3.06	75.50 ± 1.80	96.43 ± 0.72

Vin classification accuracy (%)				
Protocol	SNR = 5	SNR = 10	SNR = 20	SNR = 40
Optimized SNR 20	32.87 ± 2.77	50.17 ± 3.39	79.40 ± 2.94	98.47 ± 0.62
Optimized SNR 40	28.77 ± 2.59	44.30 ± 3.41	72.47 ± 3.14	96.33 ± 1.04
Baseline	27.73 ± 2.61	39.30 ± 2.18	62.60 ± 2.40	90.30 ± 1.83
Baseline2	28.27 ± 1.87	40.23 ± 2.80	64.60 ± 1.91	91.43 ± 1.57

*Note:* Classification accuracy for distinguishing between two cell populations (R, $V_{\text{in}}$) using Random Forest classifier with 10‐fold cross‐validation. Training and testing performed on 3000 simulated IMPULSED signals. Four protocols compared: Optimized SNR 20 (optimized for SNR = 20 conditions), Optimized SNR 40 (optimized for SNR = 40 conditions), Baseline and Baseline2 (timing setting set to that of Optimized SNR 20). Accuracy values represent mean performance across cross‐validation folds. Higher values indicate better discrimination capability.

The performance advantages of optimized protocols extended to biologically complex scenarios involving heterogeneous cell populations, as demonstrated by T‐cell infiltration simulations (Supplementary S1, Figure S5, Table S1).

#### 3.2.2 Quantification Performance

Figure 2 presents normalized RMSE for $R,V_{\text{in}},$ and $D_{\mathrm{ex}}$ across SNRs of 5, 10, 20, and 40. For all protocols and parameters, normalized RMSE decreased monotonically with increasing SNR. Across the entire noise spectrum, *Optimized SNR 20* maintained a consistent performance advantage over the *Baseline* for all parameters, demonstrating superior quantification accuracy and robustness to noise. Numerical results are shown in Table 3.

**FIGURE 2:**
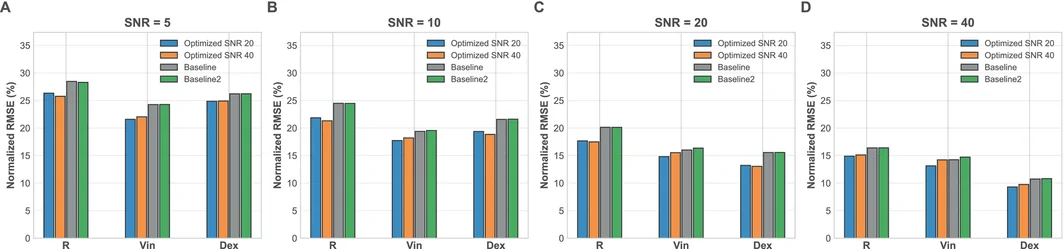
Optimized protocols reduce parameter estimation errors across noise conditions. Normalized RMSE (% of parameter maximum) for 3‐parameter fitting at SNR 5, 10, 20, and 40. Optimized protocols (blue, orange) substantially reduce RMSE compared to Baseline (gray) and Baseline2 (green).

**TABLE 3:** Quantification accuracy for biophysical parameters across protocols and noise levels.

Normalized RMSE of R				
Protocol	SNR = 5	SNR = 10	SNR = 20	SNR = 40
Optimized SNR 20	26.33	21.86	17.68	14.88
Optimized SNR 40	25.77	21.32	17.50	15.12
Baseline	28.48	24.50	20.13	16.40
Baseline2	28.30	24.48	20.12	16.41

Normalized RMSE of $V_{\text{in}}$				
Protocol	SNR = 5	SNR = 10	SNR = 20	SNR = 40
Optimized SNR 20	21.59	17.73	14.82	13.14
Optimized SNR 40	22.05	18.21	15.54	14.22
Baseline	24.27	19.39	16.01	14.25
Baseline2	24.29	19.56	16.36	14.72

Normalized RMSE of $D_{\mathrm{ex}}$				
Protocol	SNR = 5	SNR = 10	SNR = 20	SNR = 40
Optimized SNR 20	24.86	19.38	13.21	9.30
Optimized SNR 40	24.92	18.85	13.05	9.75
Baseline	26.22	21.58	15.56	10.75
Baseline2	26.23	21.62	15.57	10.82

*Note:* Normalized root‐mean‐square error (RMSE) for 3 parameter least‐squares fitting of IMPULSED model. RMSE values normalized by parameter upper bounds ($R:15\;\mathrm{\mu m},V_{\text{in}}:1.0,D_{\mathrm{ex}}:3\;\mu m^{2}/\mathrm{ms}$) and expressed as percentages for comparability across parameters. Lower values indicate more accurate parameter estimation. Quantification performed on 100 000 simulated signals with Rician noise at specified SNR levels using Trust Region Reflective algorithm.

Figure 3 shows 2D heatmaps of binned RMSE differences (*Optimized SNR 20* minus *Baseline*) for R and $V_{\text{in}},$ quantification across SNR levels, where negative regions (blue) indicate improved performance of the optimized protocol. For R quantification, the greatest improvements were observed in small‐cell regime (R < 8 μm) at higher intracellular volume fractions ($V_{\text{in}}>0.5$), with particularly strong benefits at SNRs of 10 and 20. For $V_{\text{in}}$ quantification, the optimized protocol demonstrated robust improvements across nearly the entire ($R,V_{\text{in}})$ space at SNRs of 10 and 20, with the strongest improvements occurring at larger $V_{\text{in}}$ regions.

**FIGURE 3:**
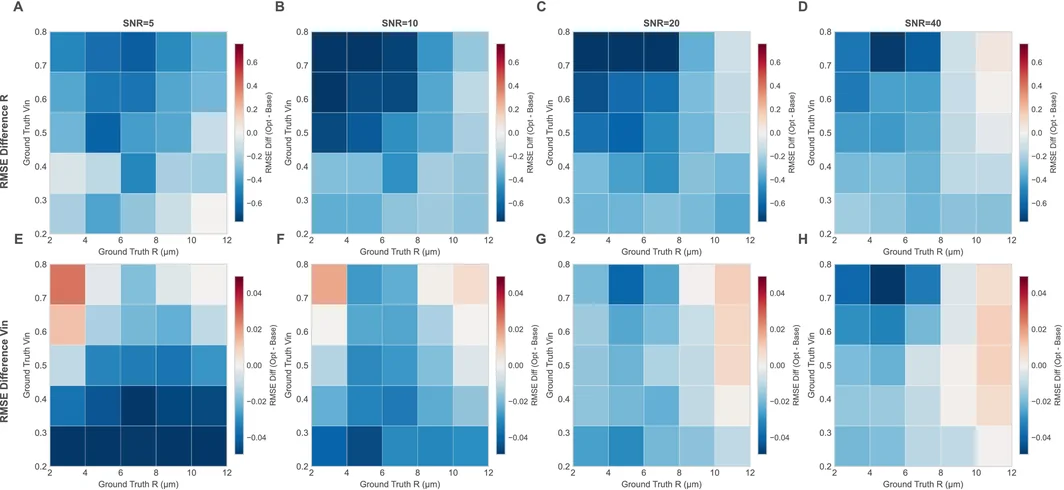
Spatial distribution of quantification improvements from optimized protocols. Difference heatmaps showing RMSE improvement (*Optimized SNR 20 − Baseline*) across biologically relevant parameter space. Row 1 shows cell radius (R) quantification; Row 2 shows intracellular volume fraction ($V_{\text{in}}$) quantification. Columns represent SNR levels 5, 10, 20, and 40. Optimized protocol demonstrates improvements (predominantly blue) across nearly all biophysical regimes and noise conditions.

Collectively, Figures 1, 2, 3 demonstrate that experimental design optimization provides higher quality data that substantially improves both classification and parameter estimation accuracy across diverse biophysical regimes and noise conditions.

### 3.3 Ablation Study

#### 3.3.1 Impact of Diffusion Time and b Values

As shown in Figure 1 the *Baseline2* protocol demonstrated slightly improved performance for classification of both R and $V_{\text{in}}$, especially for SNR≥10. However, it remained inferior to *Optimized SNR 20* across all SNR levels. For quantification (Figure 2), *Baseline2* achieved similar RMSE compared with the *Baseline* across all SNRs for all parameters.

#### 3.3.2 Impact of SNR Level

As shown in Figure 1 the *Optimized SNR 40* protocol demonstrated improved performance comparing with the *Baseline* and a worse performance comparing with the *Optimized SNR 20* across all SNRs. For quantification (Figure 2), the *Optimized SNR 40* protocol achieved similar RMSE as *Optimized SNR 20*; both optimized protocols substantially outperformed the *Baseline* and *Baseline2* protocols across all SNRs, confirming the robustness and generalizability of Bayesian experimental design optimization for IMPULSED.

### 3.4 Impact of Assumptions on Optimized Acquisition Protocols

Optimized pulse parameters across four SNR levels and nine prior distributions are summarized in Tables 4 and 5. The results reveal SNR‐dependent and prior‐dependent patterns for both *b*‐value distributions and diffusion timings across pulses.

**TABLE 4:** Pulse sequence parameters optimized for different noise conditions.

Prior SNR	Sequence	δ/Δ(ms)	$t_{\text{diff}}\;\left(\mathrm{ms}\right)$	b‐value $\left(s/mm^{2}\right)$
SNR 5	PGSE	34/58	46.67	1350(1), 1853(1), 1962(1), 2000(5)
	OGSEn1	41/51	10.25	1190(1), 1220(4)
	OGSEn2	N/A		
SNR 10	PGSE	32/60	49.33	798(1), 1004(1), 1316(1), 2000(4)
	OGSEn1	41/51	10.25	1105(1), 1109(1), 1194(1), 1220(3)
	OGSEn2	N/A		
SNR 20	PGSE	33/59	48.00	688(1), 800(1), 829(1), 931(1), 2000(4)
	OGSEn1	41/51	10.25	1220(5)
	OGSEn2	N/A		
SNR 40	PGSE	26/62	53.33	583(1), 710(1), 767(1), 871(1), 2000(3)
	OGSEn1	41/51	10.25	1220(4)
	OGSEn2	41/51	5.125	298(2)

*Note:* Optimized protocol parameters obtained via Bayesian optimization under UR‐UVin prior distribution at four SNR levels (5, 10, 20, 40). All optimizations used identical prior assumptions and scanner hardware constraints, varying only the noise level. Each optimization ran for 500 iterations to maximize Expected Information Gain. Protocol adaptations reflect information‐theoretic trade‐offs between signal strength and diffusion weighting under different noise regimes. Numbers in parentheses indicate the number of acquisitions at the corresponding *b*‐value. The maximum OGSEn2 *b*‐value (298 s/mm^2^) differs slightly from the baseline protocol (300 s/mm^2^) due to floating‐point precision and minor variations in achievable gradient strength.

Abbreviations: δ, gradient pulse duration; Δ, time interval between diffusion encoding gradients; $t_{\text{diff}}$, effective diffusion time; OGSE, oscillating gradient spin echo with oscillation number *n*; PGSE, pulsed gradient spin echo.

**TABLE 5:** Pulse sequence parameters optimized for different biological assumptions.

Prior type	Sequence	δ/Δ(ms)	$t_{\text{diff}}\;\left(\mathrm{ms}\right)$	b‐value $\left(s/mm^{2}\right)$
UR‐SVin	PGSE	26/66	57.33	716(1), 745(1), 768(1), 916(1), 2000(4)
	OGSEn1	41/51	10.25	1220(5)
	OGSEn2	N/A		
UR‐LVin	PGSE	26/66	57.33	582(1), 749(1), 801(1), 848(1), 1905(1), 2000(3)
	OGSEn1	41/51	10.25	1124(1), 1220(4)
	OGSEn2	N/A		
SR‐SVin	PGSE	10/82	78.67	673(1), 683(1), 789(1), 1003(1), 2000(4)
	OGSEn1	41/51	10.25	1118(1), 1220(4)
	OGSEn2	N/A		
SR‐UVin	PGSE	10/82	78.67	694(1), 707(1), 819(1), 968(1), 977(1), 2000(3)
	OGSEn1	41/51	10.25	1154(1), 1220(3)
	OGSEn2	41/51	5.125	298(1)
SR‐LVin	PGSE	24/67	59.00	734(1), 832(1), 851(1), 1019(1), 2000(4)
	OGSEn1	41/51	10.25	1220(5)
	OGSEn2	N/A		
LR‐SVin	PGSE	25/60	51.77	705(1), 745(1), 765(1), 989(1), 2000(4)
	OGSEn1	40/52	10.25	934(1), 1004(1), 1093(1), 1098(1), 1131(1)
	OGSEn2	N/A		
LR‐UVin	PGSE	35/57	45.33	712(1), 753(1), 780(1), 841(1), 2000(4)
	OGSEn1	41/51	10.25	977(1), 1066(1), 1191(1), 1220(2)
	OGSEn2	N/A		
LR‐LVin	PGSE	29/63	53.34	754(1), 773(1), 790(1), 866(1), 1998(1), 2000(3)
	OGSEn1	40/52	10.25	1131(5)
	OGSEn2	N/A		

*Note:* Optimized protocol parameters obtained via Bayesian optimization under SNR = 20 noise assumption with nine different prior distributions over cell parameters. Prior distributions represent combinations of R biases (Uniform, Small, Large) and $V_{\text{in}}$ biases (Uniform, Small, Large). UR‐UVin, uniform R and $V_{\text{in}}$; SR‐UVin, small R‐biased; LR‐UVin, large R‐biased; UR‐SVin, small $V_{\text{in}}$‐biased; and so forth. Numbers in parentheses indicate the number of acquisitions at the corresponding *b*‐value. The maximum OGSEn2 *b*‐value (298 s/mm^2^) differs slightly from the baseline protocol (300 s/mm^2^) due to floating‐point precision and minor variations in achievable gradient strength.

Abbreviations: δ, gradient pulse duration; Δ, time interval between two diffusion pulses; $t_{\text{diff}}$, effective diffusion time; OGSE, oscillating gradient spin echo with oscillation number *n*; PGSE, pulsed gradient spin echo.

For PGSE acquisitions, *b*‐value sampling was concentrated at high *b*‐value region approaching the maximum achievable value (2000 s/mm^2^), with multiple acquisitions at the maximum *b*‐value. As SNR increased, sampling expanded toward intermediate‐to‐high *b*‐value, a pattern consistently observed across most prior distributions at SNR 20, indicating robustness to prior assumptions. The optimized PGSE diffusion time also showed an increasing trend with SNR, from $t_{\text{diff}}=46.67$ ms at low SNR to $t_{\text{diff}}=53.33$ ms at higher SNR level. Notably, $t_{\text{diff}}$ of PGSE exhibited sensitivity to prior distributions, with longer $t_{\text{diff}}$ favored under small‐cell‐biased priors. For OGSEn1 acquisitions, optimized protocols consistently concentrated measurements at the maximum achievable *b*‐value (∼1220 s/mm^2^) across all SNR levels and prior distributions. The $t_{\text{diff}}$ for OGSEn1 remained constant regardless of both SNR and prior distribution, consistently maintaining the maximum gradient duration (δ=41ms). OGSEn2 acquisitions were largely excluded from optimized protocols. At lower SNRs (5, 10, and 20), OGSEn2 was absent from all optimized protocols. It appeared only rarely across prior distributions at SNR 20 and emerged consistently only at the highest SNR (40).

### 3.5 In‐Vivo Animal Patient Study: Improved Parameter Estimation Stability With Optimized Protocol

Visual comparison of parameter maps from *Baseline* and *Optimized SNR 20* were shown in Figure 4. Parameter maps derived from the *Optimized SNR 20* exhibited smoother spatial distributions across all estimations ($R,V_{\text{in}},D_{\mathrm{ex}}$) compared to the *Baseline*. The optimized protocol substantially reduced the occurrence of extreme pixel values compared to *Baseline*. Extreme pixels, where estimation reached fitting boundaries, decreased from 33.3% (*Baseline*) to 7.9% (*Optimized*). Parameter histograms (Figure 4I–K) showed: R=6.70±2.41μm (*Optimized*) versus 7.66 ± 3.15 μm (*Baseline*); $V_{\text{in}}=0.29\pm 0.10$ versus 0.36 ± 0.14; $D_{\mathrm{ex}}=1.28\pm 0.38$ versus 1.49 ± 0.50 ${\mathrm{\mu m}}^{2}/\mathrm{ms}$.

**FIGURE 4:**
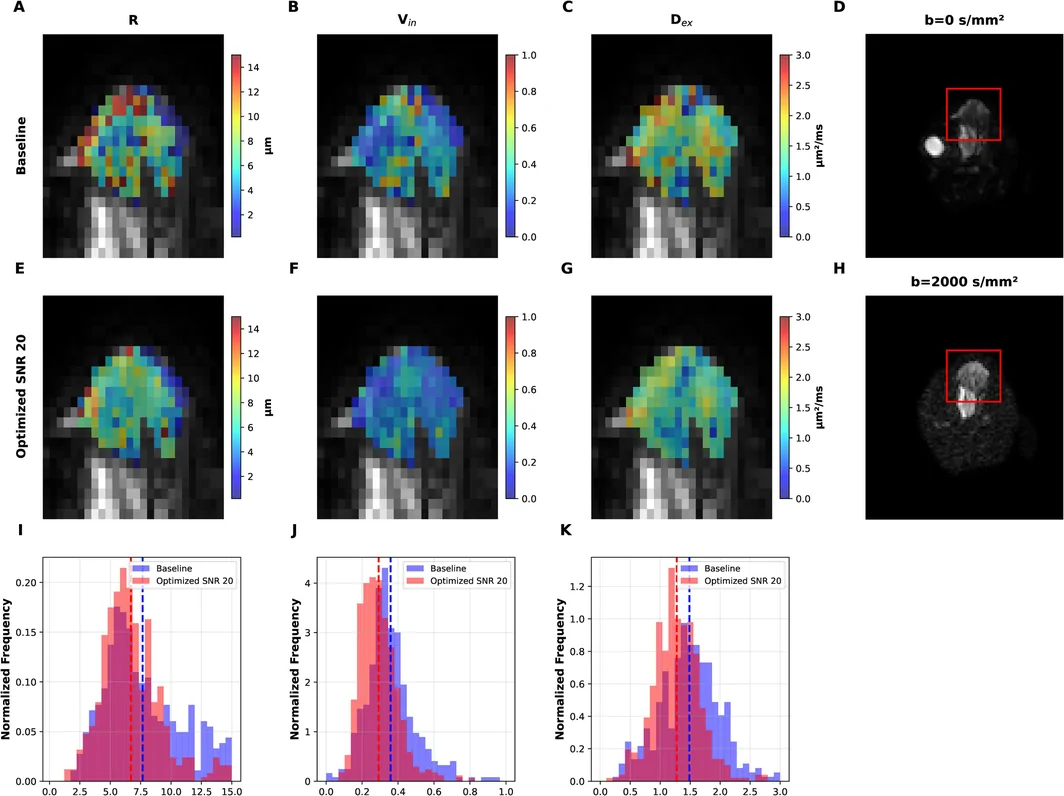
In vivo comparison of baseline and optimized protocols for IMPULSED parameter estimation in canine tissue. Parameter maps of cell radius (R), intracellular volume fraction ($V_{\text{in}}$), and extracellular diffusivity ($D_{\mathrm{ex}}$) estimated from a canine patient undergoing radiation therapy. (A–C) *Baseline* protocol results overlaid on *b* = 0 $s/{\mathrm{mm}}^{2}$ reference image. (D) Full field‐of‐view *b* = 0 s/mm^2^ image with red box indicating the region of interest. (E–G) *Optimized SNR 20* protocol results overlaid on *b* = 0 $s/{\mathrm{mm}}^{2}$ reference image. (H) Full field‐of‐view *b* = 2000 $s/{\mathrm{mm}}^{2}$ image with red box indicating the region of interest. (I–K) Histograms comparing parameter distributions between *Baseline* (blue) and *Optimized* (red) protocols for the entire tumor volume (excluding extreme pixels that reached fitting boundaries). Dashed lines indicate mean values. All parameter maps were estimated using nonlinear least‐squares fitting with identical boundary constraints ($R:0.2-15\;\mathrm{\mu m},V_{\text{in}}:0-1,D_{\mathrm{ex}}:0-3.0\;{\mathrm{\mu m}}^{2}/\mathrm{ms}$). The optimized protocol yields visibly smoother parameter maps with reduced pixel‐to‐pixel variability and fewer extreme values, demonstrating improved estimation stability compared to the baseline protocol.

Consistently superior performance of the optimized protocol was confirmed in a follow‐up scan 1 month later, showing similar reductions in extreme pixels and improved parameter stability (Supplementary S3, Figures S6 and S7).

## 4 Discussion

### 4.1 Insights Into Optimized Acquisition Strategies

Across optimization scenarios, the optimized protocols consistently concentrated measurements at high *b*‐values. This pattern indicates that IMPULSED signals, particularly those acquired with OGSEn1 and OGSEn2, are highly sensitive to measurement noise, such that observed signal decay under clinical SNR conditions is effectively dominated by mono‐exponential behavior. Consequently, only a subset of model parameters can be reliably identified in vivo. At SNR = 40, OGSEn2 acquisitions with shorter $t_{\text{diff}}$ began to emerge, indicating partial recovery of additional parameter sensitivity as noise decreases. In contrast, OGSEn2 was systematically excluded at lower SNR levels. This reflects a trade‐off between the sensitivity of high‐frequency oscillating gradients to short‐range cellular structure and their limited achievable *b* values under hardware constraints. The maximum gradient strength on clinical 3T scanners restricts the achievable *b*‐value for OGSEn2 to be below 300 s/mm^2^, resulting in limited signal contrast (i.e., small signal difference between *b* = 0 and *b* = 300 acquisitions) compared to PGSE and OGSEn1 sequences. Under low SNR, the already‐weak signal decay produced by low‐b OGSEn2 acquisitions falls below the noise variance, contributing measurement variance rather than biophysical information. Concentrating acquisitions at high *b* values therefore represents a pragmatic strategy to optimized protocols prioritize reliable estimation of identifiable parameters rather than attempting to exhaustively characterize all aspects of the model.

The optimized diffusion timing parameters also exhibited systematic SNR‐dependent patterns. As SNR increased, the optimized protocols expanded the range of effective diffusion times. This behavior is consistent with information‐theoretic expectations: higher measurement precision allows acquisition strategies to exploit a broader range of contrast mechanisms.

An unexpected relationship was observed between the prior distribution and the optimized PGSE diffusion time. Priors biased toward smaller cell sizes resulted in longer optimized diffusion times, contrary to the conventional expectation that longer diffusion times primarily enhance sensitivity to larger structures. This effect may arise from interactions between the multi‐compartment structure of the IMPULSED model and the information content at different diffusion timescales. One potential explanation is that for small cells, longer diffusion times may enhance the contrast between intracellular and extracellular compartments, thereby improving parameter identifiability despite probing spatial scales larger than the cell dimensions.

### 4.2 Relative Contributions of Acquisition Parameters

The ablation study using *Baseline2* protocol provided insights into the relative contributions of diffusion timing and *b*‐value optimization. *Baseline2* adopted the optimized diffusion times from the *Optimized SNR 20* protocol while maintaining the *b*‐value distribution of *Baseline*. Although Baseline2 showed slight improvement over the Baseline protocol, it remained substantially inferior to the fully optimized protocol for classification tasks (Figure 1), indicating that the *b*‐value distribution plays a more dominant role than diffusion timing in determining measurement quality.

The influence of assumed SNR during optimization was evaluated by comparing *Optimized SNR 40* and *Optimized SNR 20* protocols. Despite structural differences between the two protocols, their performance across the tested SNR range was similar. This suggests that the EIG landscape contains multiple near‐optimal solutions with distinct acquisition configurations that achieve comparable performance.

### 4.3 Practical Implications for Clinical Protocol Design

These results provide practical guidance for clinical protocol design. For typical clinical SNR levels (10–20), acquisition resources should primarily target PGSE and OGSEn1 measurements with intermediate‐to‐high *b* values, while OGSEn2 contributes limited additional information and can be omitted to improve efficiency. OGSEn2 may only become beneficial at higher SNR (> 40), where measurement precision allows the additional short‐diffusion‐time information to be effectively utilized. The robustness of optimized protocols' performance across SNR levels further suggests that a protocol optimized for SNR 20 can be reliably deployed across a range of clinical imaging conditions. Furthermore, the prior‐dependent modulation of optimal PGSE diffusion times suggests that in settings where strong prior anatomical knowledge is available, protocol customization based on expected tissue characteristics could yield additional performance gains.

Note that in Figure 1, the *Optimized* protocols consistently outperformed the *Baseline* protocol, with the largest improvements observed at intermediate SNRs of 10 and 20. At a low SNR of 5, the performance advantage diminished because measurements were dominated by noise. Conversely, at a high SNR of 40, the *Baseline* protocol already achieved good performance, reducing the benefit of optimization. This SNR‐dependent performance pattern indicates our optimized protocols will benefit the clinical application of the IMPULSED model, where a typical SNR ranges at 10–20.

### 4.4 Quantification Challenges of the IMPULSED Model

The IMPULSED model involves a highly nonlinear mapping between microstructural parameters and diffusion signals, resulting in an ill‐conditioned inverse problem in which different parameter combinations can produce similar signal decays [7, 48]. This limitation is reflected in the spatially heterogeneous quantification errors observed in Figure S4. For R quantification, the largest RMSE occurred in the small‐cell (R < 4 μm) and large‐cell (R>10μm) regimes at low SNR, with errors remaining relatively elevated for large cells even at higher SNR. This likely reflects a mismatch between the diffusion times probed and the characteristic diffusion length scales of large cells. For $V_{\text{in}}$ quantification, estimation was most challenging at high $V_{\text{in}}$ (> 0.7) combined with small and large cell radii for both protocols, likely due to reduced contrast between intra‐ and extracellular diffusion components. Despite these intrinsic limitations, the optimized protocol consistently reduced RMSE compared with the baseline protocol.

### 4.5 In‐Vivo Validation

In vivo animal patient feasibility study demonstrated improved parameter map stability with the optimized protocol. The resulting maps exhibited smoother spatial patterns and fewer extreme outliers than those obtained with the baseline protocol, consistent with the expected spatial continuity of biological tissue properties. These improvements were achieved without increasing total acquisition time, supporting the feasibility of deploying the optimized protocol in clinical IMPULSED studies.

### 4.6 Generality and Limitations of the Framework

A key feature of our Bayesian experimental design framework is its foundation in information theory. Rather than optimizing task‐specific metrics such as classification accuracy or RMSE for a particular fitting algorithm, the EIG objective directly quantifies the reduction in parameter uncertainty achievable from a given acquisition protocol. By maximizing the information content of acquired measurements, the optimized protocols support diverse analysis strategies, including classification, regression, and parameter estimation using different inference methods [9, 13, 14]. Consequently, the optimization remains independent of downstream analysis methods. This flexibility is particularly valuable for IMPULSED dMRI cytometry, where optimal quantification strategies remain an active area of investigation, and ongoing work includes neural network‐based [9, 14], traditional least‐squares fitting, and Bayesian inference methods for parameter estimation [13]. The framework also naturally incorporates nonlinear signal–parameter relationships and realistic noise models without requiring analytical tractability, since EIG is evaluated using Monte Carlo sampling over parameter priors and measurement noise. In contrast, traditional methods like CRLB have two fundamental drawbacks in our application. First, CRLB requires inversion of the Fisher information matrix, which can become singular or near‐singular matrix for sloppy models such as IMPULSED. More importantly, CRLB is only a local, linearization‐based approximation, and the scalar objectives typically constructed from it, such as the trace, further discard the off‐diagonal parameter‐covariance information. EIG, in contrast, is a global information‐theoretic quantity that captures the full nonlinear likelihood (Supplementary S4). Further, although demonstrated here for the IMPULSED model, the same Bayesian optimization framework can be readily applied to other quantitative MRI techniques that rely on forward signal models linking tissue properties to measurements. Extension to other models, such as Vascular Extracellular and Restricted Diffusion for Cytometry in Tumors (VERDICT) [49], represents an important direction for future work.

Several limitations should be acknowledged. First, the optimization assumes that the IMPULSED model accurately describes the underlying diffusion physics. Model inaccuracies, such as neglected membrane permeability, cellular shape anisotropy, or additional diffusion compartments, may bias the information content targeted by the optimization. Second, while the optimized protocols showed robustness to moderate prior misspecification, their performance may vary under substantially different biological conditions. Finally, despite the encouraging in vivo results, the evaluation was limited to one canine patient due to scan time and anesthesia constraints. While a second session confirmed improvements, the 1‐month interval prevented repeatability analysis. Future work includes multi‐subject validation with same‐day repeatability and longitudinal cancer patient studies monitoring treatment‐induced parameter changes. These ongoing studies will validate the method's sensitivity to treatment‐induced microstructural changes and assess the optimized protocol's ability to detect clinically relevant biological alterations over time.

## 5 Conclusion

We developed and validated a Bayesian experimental design optimization framework for IMPULSED dMRI cytometry. Information‐theoretic optimization of pulse sequence parameters yields more informative data acquisition, improving both classification discriminability and parameter estimation accuracy versus conventional acquisition strategies. Systematic analysis of optimization convergence under varying assumptions provides insights into IMPULSED model behavior under clinical SNR, providing guidance for implementing dMRI cytometry.

## Funding

This work was supported by VROC Seed Funding 2024, UT Southwestern Medical Center; National Cancer Institute, R37CA214639.

## Conflicts of Interest

The authors declare no conflicts of interest.

## Supporting information


**Supplementary S1:** T‐cell infiltration classification study.
**Supplementary S2:** Convergence analysis and protocol equivalence.
**Supplementary S3:** Longitudinal result analysis: parameter changes following radiation treatment.
**Supplementary S4:** Comparison with Cramér‐Rao Lower Bound‐based optimization.
**Figure S1:** Illustration of diffusion‐weighted pulse sequences used in IMPULSED acquisition protocols. Schematic diagrams showing three pulse sequence types: PGSE (pulsed gradient spin echo), OGSEn1 (oscillating gradient spin echo with one oscillation period), and OGSEn2 (oscillating gradient spin echo with two oscillation periods). Each sequence shows the RF pulses (90° excitation and 180° refocusing), diffusion‐weighting gradients (trapezoid waveforms), and signal acquisition (ACQ).
**Figure S2:** Prior distributions for Bayesian experimental design optimization. Nine prior distributions representing combinations of R biases (Uniform, Small, Large) and $V_{\text{in}}$ biases (Uniform, Small, Large). Each panel shows marginal distributions for $R,D_{\text{in}},V_{\text{in}},D_{\mathrm{ex}}$, and $\beta_{\mathrm{ex}}$ from 100 000 samples.
**Figure S3:** Convergence of Bayesian optimization across conditions. Maximum EIG over 500 iterations for 12 optimization cases. Row 1 shows UR‐UVin prior at SNR 5, 10, 20, 40, demonstrating strong SNR dependence (final EIG: 1.05 to 4.90). Rows 2–3 show eight additional priors at SNR = 20. All optimizations converge within 100–200 iterations to similar EIG values (2.95–3.33), indicating noise level dominates over prior shape.
**Figure S4:** Direct comparison of absolute RMSE values between optimized and baseline protocols. Absolute RMSE heatmaps for direct protocol comparison. Rows 1–2 show cell radius (R) quantification for Optimized SNR 20 and Baseline protocols, respectively. Rows 3–4 show intracellular volume fraction ($V_{\text{in}}$) quantification for Optimized SNR 20 and Baseline protocols, respectively. Columns represent SNR levels 5, 10, 20, and 40. Color intensity indicates normalized RMSE (% of parameter maximum). Comparison between row pairs reveals systematic RMSE reduction with optimized protocols across the parameter space.
**Figure S5:** Optimized protocols improve T‐cell infiltration classification across noise conditions. Random Forest classification accuracy at SNR levels 5, 10, 20, and 40 for four protocols: *Optimized SNR 20* (blue), *Optimized SNR 40* (orange), *Baseline2* (green), and *Baseline* (grey). Signals generated from weighted mixtures of two cell populations (tumor cells R=10μm; T‐cells R=7μm) sharing identical diffusion parameters ($V_{\text{in}}=0.7,D_{\text{in}}=1.1\;{\mathrm{\mu m}}^{2}/\mathrm{ms},D_{\mathrm{ex}}=0.7\;{\mathrm{\mu m}}^{2}/\mathrm{ms},\beta_{\mathrm{ex}}=3.0\;{\mathrm{\mu m}}^{2}$) at infiltration levels 0%, 15%, and 30%. Optimized protocols achieve higher accuracy across all noise conditions. PCA of signal vectors with varying infiltration levels illustrated enhanced class separability. The *Optimized SNR 20* protocol produced substantially improved separation between classes compared with the Baseline protocol, yielding distinct and well‐separated clusters in the dominant principal component space.
**Figure S6:** In vivo comparison of baseline and optimized protocols for IMPULSED parameter estimation in canine tumor. Parameter maps of cell radius (R), intracellular volume fraction ($V_{\text{in}}$), and extracellular diffusivity ($D_{\mathrm{ex}}$) estimated from the same canine patient undergoing radiation therapy as shown in Figure 4. The acquisition was performed after the first treatment fraction, with one‐month interval between the previous acquisition. (A–C) *Baseline* protocol results overlaid on *b* = 0 $s/{\mathrm{mm}}^{2}$ reference image. (D) Full field‐of‐view *b* = 0 s/mm^2^ image with red box indicating the region of interest. (E–G) *Optimized SNR 20* protocol results overlaid on *b* = 0 $s/{\mathrm{mm}}^{2}$ reference image. (H) Full field‐of‐view *b* = 2000 $s/{\mathrm{mm}}^{2}$ image with red box indicating the region of interest. (I–K) Histograms comparing parameter distributions between *Baseline* (blue) and *Optimized* (red) protocols for the entire tumor volume (excluding extreme pixels that reached fitting boundaries). Dashed lines indicate mean values. All parameter maps were estimated using nonlinear least‐squares fitting with identical boundary constraints ($R:0.2-15\;\mathrm{\mu m},V_{\text{in}}:0-1,D_{\mathrm{ex}}:0-3.0\;{\mathrm{\mu m}}^{2}/\mathrm{ms}$). The optimized protocol yields visibly smoother parameter maps with reduced pixel‐to‐pixel variability and fewer extreme values, demonstrating improved estimation stability compared to the baseline protocol.
**Figure S7:** In vivo visualization of IMPULSED parameter evolution after one treatment fraction in canine tumor using the optimized protocol. Histograms demonstrate parameter distribution shift before and after radiation treatment with 1 month interval in the same canine patient. All histograms display data from the entire tumor volume, excluding extreme pixels that reached fitting boundary limits. Dashed lines indicate the mean value for cell radius R, intracellular volume fraction $V_{\text{in}}$, and extracellular diffusion coefficient $D_{\mathrm{ex}}$ distribution obtained using the *Optimized SNR 20* protocol.
**Table S1:** Random Forest classification accuracy for T‐cell infiltration detection. Classification accuracy for distinguishing T‐cell infiltration levels (0%, 15%, 30%) using Random Forest classifier with 10‐fold cross‐validation. Signals generated from weighted mixtures of tumor cells (R=10μm) and T‐cells (R=7μm) with identical diffusion parameters ($V_{\text{in}}=0.7,D_{\text{in}}=1.1\;{\mathrm{\mu m}}^{2}/\mathrm{ms},D_{\mathrm{ex}}=0.7\;{\mathrm{\mu m}}^{2}/\mathrm{ms},\beta_{\mathrm{ex}}=3.0\;{\mathrm{\mu m}}^{2}$). Accuracy values represent mean performance across cross‐validation folds. Higher values indicate better discrimination capability.
**Table S2:** EIG‐ranked protocols re‐scored under the CRLB objective. Eight representative protocols were selected from the 800 protocols visited by the EIG‐based Bayesian‐optimization (UR‐UVin, SNR = 20), corresponding to EIG‐rank percentiles #1, 2%, 5%, 10%, 15%, 20%, 30%, and 50%. Each protocol was re‐scored under the CRLB score. Note that higher = better for both EIG and CRLB score.
**Table S3:** EIG‐ranked protocols re‐scored under the CRLB objective. The CRLB protocol were obtained via Bayesian optimization maximizing CRLB score under UR‐UVin prior distribution with SNR = 20.
**Table S4:** Classification and quantification performance comparison of CRLB score based optimization with Baseline and Optimized SNR 20. (a) Random‐forest R and $V_{\text{in}}$ classification accuracy (mean ± standard deviation over 10‐fold cross‐validation). (b) RMSE values normalized by parameter upper bounds ($R:15\;\mathrm{\mu m},D_{\text{in}}:3\;{\mathrm{\mu m}}^{2}/\mathrm{ms},V_{\text{in}}:1.0,D_{\mathrm{ex}}:3\;{\mathrm{\mu m}}^{2}/\mathrm{ms},\beta_{\mathrm{ex}}:10$). Quantification are all based on the 3‐parameter LSQ fit for R, $V_{\text{in}}$, and $D_{\mathrm{ex}}$, with $D_{\text{in}}=1.58\;{\mathrm{\mu m}}^{2}/\mathrm{ms}$ and $\beta_{\mathrm{ex}}=2\;{\mathrm{\mu m}}^{2}$ fixed.

## Data Availability

The data that support the findings of this study are available on request from the corresponding author. The data are not publicly available due to privacy or ethical restrictions.
