# Supplementary material for: Optimizing IMPULSED Acquisition Protocols for Clinical 3T Scanners Through Bayesian Experimental Design
Source: Magn Reson Med. 2026 Sep 14;97(1):100–13. doi: 10.1002/mrm.70579 (PMC13746092; doi:10.1002/mrm.70579)
Supplement: Supplementary file 1 [file MRM-97-100-s001.docx]

**S1. T-cell Infiltration Classification Study**

To evaluate protocol performance under biologically realistic heterogeneity, an additional classification study was performed modeling T-cell infiltration into tumor tissue. Signals were generated from weighted mixtures of two cell populations: tumor cells ($R=10 \mu m$) and T-cells ($R=7 \mu m$), both sharing identical diffusion parameters ($V_{in}=0.7, D_{in}=1.1 \mu m^{2}/ms, D_{ex}=0.7 \mu m^{2}/ms, \beta_{ex}=3.0 \mu m^{2}$). Three infiltration levels were simulated (0%, 15%, 30%), representing the volume fraction of T-cells relative to total cells. Noisy signals were generated and classification was performed following the same method described above.

For the T-cell infiltration simulation (**Figure S5**, **Table S1**), the Optimized SNR 20 protocol consistently achieved higher classification accuracy than the *Baseline* protocol across all SNR levels tested. PCA of signal vectors illustrated enhanced class separability, with the *Optimized SNR 20* protocol producing substantially improved separation between infiltration levels compared with the Baseline protocol (e.g. classification accuracy of 78.47 ± 3.04% by *Optimized SNR 20* vs. 63.67 ± 3.09% by *Baseline*). These results demonstrate that the performance advantages of optimized protocols extend to biologically complex scenarios involving heterogeneous cell populations.

**S2. Convergence Analysis and Protocol Equivalence**

The stochastic nature of Monte Carlo-based EIG evaluation can result in optimized protocols with similar b-values in close proximity (e.g., b = 1998 and 2000 s/mm² for PGSE in the LR-LVin prior). Convergence plots (**Figure S3**) confirm that the optimization reached a plateau, indicating these similar parameters represent nearly equivalent protocols with indistinguishable EIG values and signal measurements. While further iterations with larger Monte Carlo sample sizes might yield more uniform b-value spacing, such differences are negligible in practice compared to the larger variations in optimal protocols for different prior distributions (**Tables 4-5**).

**S3. Longitudinal Result Analysis: Parameter Changes Following Radiation Treatment**

Consistently superior performance of the optimized protocol was also observed in an additional scan session from the same patient acquired at a subsequence radiation treatment fraction one month later (**Figure S6**). Similarly, in this session, the *Baseline* protocol yielded 51.7% extreme pixels, whereas the *Optimized* protocol yielded only 14.3% extreme pixels, representing a 72.3% reduction. The comparison of parameters before and after the treatment fraction, quantified using the *Optimized* protocol, is shown in **Figure S7**. $R{, V}_{in}, \mathrm{and}D_{ex}$ demonstrated changes of -7.6%, +0.7%, and -8.1%, respectively. These findings may reflect treatment-induced biological changes associated with apoptosis, leading to reduced mean cell size. In combination with the relatively unchanged$V_{in}$, the results suggest increased cell size heterogeneity and altered extracellular microstructure.

**S4. Comparison with Cramér-Rao Lower Bound-Based Optimization**

The choice of EIG over alternative optimization objectives warrants discussion. Cramer-Rao Lower Bound (CRLB) based approaches have been successfully applied to experimental design optimization for simpler diffusion MRI models, including Diffusion Tensor Imaging (DTI) and multi-compartment models(26-29). However, CRLB has two fundamental drawbacks that limit its suitability for IMPULSED.

First, CRLB requires inversion of the Fisher Information Matrix (FIM), which becomes rank-deficient for sloppy models like IMPULSED where multiple parameter combinations produce nearly indistinguishable signals(7,48). A model is called sloppy when the eigenvalue spectrum of its Fisher Information Matrix spans many orders of magnitude, making some parameters highly correlated and parameter estimation challenging. Evaluating FIM conditioning for the Baseline protocol across the same grid of parameter combinations used for EIG computation revealed that approximately 79% yielded condition numbers exceeding 10^6^, with median condition number being 2.0×10^^^6, indicating substantial numerical ill-conditioning.  Because this fraction of ill-conditioned prior samples must be evaluated at every candidate design considered during Bayesian optimization, the CRLB target function is numerically unresolvable at many points along the optimization trajectory.

Second but more important, CRLB is only a local, linearization-based approximation to the estimator covariance at a specific ground-truth parameter value, whereas experimental design optimization requires evaluating protocol quality over a broad prior of physiologically plausible parameters. Moreover, the scalar objectives typically constructed from CRLB further collapse the local FIM to a diagonal sum, discarding the off-diagonal parameter-covariance information contained even within that local approximation.

In contrast, EIG quantifies expected entropy reduction across the entire parameter space, providing a global uncertainty measure unaffected by local FIM ill-conditioning and does not rely on any local linearization or scalar-projection step. This makes EIG particularly suitable for complex biophysical models like IMPULSED.

**Definition of the range-normalized CRLB score**

For a given candidate protocol $d$ and a set of parameters $\theta$, the Fisher Information Matrix $FIM(\theta, d)$ was computed by central finite differences of the IMPULSED forward signal (step size $10⁻⁴$) given noise ($\sigma= 1/20, SNR = 20$). To ensure numerical invertibility, the $FIM$ was regularized as $FIM + {10}^{-8}\times I$, where $I$ is a unit matrix, before inversion. The diagonal of the inverse FIM, $FIM^{-1}\left( \theta,d \right)_{pp}$, gives the Cramér–Rao lower bounds of the per-parameter estimator variances. Their square roots were normalized by each parameter's plausible range $\Delta p$ and summed across the five IMPULSED parameters to yield an uncertainty given certain $\theta$ and $d$, with $\Delta R = 14.8 \mu m$, $\Delta D_{in} = 3 \mu m^{2}/ms$, $\Delta V_{in} = 1$, $\Delta D_{ex} = 3 \mu m^{2}/ms$, $\Delta\beta_{ex} = 10$ $\mu m^{2}$. Averaging this quantity over the 2304-point prior grid $\{\theta\}$ used for EIG evaluation and taking its negative base-10 logarithm defined the per-protocol CRLB score:

$$score\left( d \right)= -\log_{10} \frac{1}{2034}\sum_{\theta} \sum_{p} \sqrt{FIM^{-1}\left( \theta,d \right)_{pp}} / \Delta p,$$

Eq. S1

If the regularized inverse still failed during the process of Bayesian Optimization, a large penalty 10¹² will be assigned.

.

**Correlation of the Range-Normalized CRLB Score with EIG**

To assess whether the CRLB and EIG objectives rank the visited candidate protocols consistently, we took the 800 protocols visited during the EIG-based Bayesian Optimization (BO) run (UR-SVin, SNR=20, 300 initial + 500 iterations), ranked them by descending EIG, and selected representative protocols at the 1st, 2%, 5%, 10%, 15%, 20%, 30%, and 50% percentile ranks. Each selected protocol was then re-scored with the CRLB score defined above; results are reported in Table S2. The two objectives disagree strongly: the top-EIG protocol (EIG = 3.388) has one of the worst CRLB scores in the tested subset (CRLB score = −3.172), while the best CRLB score in the tested subset (CRLB score = −1.198) is achieved by the protocol at the 50% EIG percentile (EIG = 3.168). The CRLB score is therefore not correlated with EIG within the visited set, consistent with the conceptual difference between the two objectives, that CRLB is a local approximation to the estimator variance at specific ground-truth parameter values, whereas EIG is a global information-theoretic reduction in posterior entropy averaged over the entire prior and the sampling distribution of the measurement.

**CRLB-based Bayesian optimization**

Keeping every other setting identical to the EIG-based BO run, we replaced the EIG objective with CRLB score defined above. The resulting CRLB-based optimized protocol is listed in **Table S3**. We evaluated it at SNR = 20 on the same classification and quantification procedures described in **Section 2.5**, and compared its performance to the **Baseline** and **Optimized SNR 20** in **Table S3**. It tracks the **Baseline** very closely on both benchmarks and remains clearly worse than the **Optimized SNR 20**, confirming that CRLB-driven Bayesian optimization does not yield downstream classification or quantification gains for IMPULSED model experimental design optimization.

**Note:** All references cited in supplementary materials are numbered according to the main manuscript reference list.

**Figure S1: Illustration of diffusion-weighted pulse sequences used in IMPULSED acquisition protocols.**

Schematic diagrams showing three pulse sequence types: PGSE (pulsed gradient spin echo), OGSEn1 (oscillating gradient spin echo with one oscillation period), and OGSEn2 (oscillating gradient spin echo with two oscillation periods). Each sequence shows the RF pulses (90° excitation and 180° refocusing), diffusion-weighting gradients (trapezoid waveforms), and signal acquisition (ACQ).

**Figure S2: Prior distributions for Bayesian experimental design optimization.**

Nine prior distributions representing combinations of $R$ biases (Uniform, Small, Large) and $V_{in}$ biases (Uniform, Small, Large). Each panel shows marginal distributions for $R, D_{in}, V_{in}, D_{ex}$, and $\beta_{ex}$ from 100,000 samples.

**Figure S3: Convergence of Bayesian optimization across conditions.**

Maximum EIG over 500 iterations for 12 optimization cases. Row 1 shows UR-UVin prior at SNR 5, 10, 20, 40, demonstrating strong SNR dependence (final EIG: 1.05 to 4.90). Rows 2-3 show eight additional priors at SNR=20. All optimizations converge within 100-200 iterations to similar EIG values (2.95-3.33), indicating noise level dominates over prior shape.

**Figure S4: Direct comparison of absolute RMSE values between optimized and baseline protocols.**

Absolute RMSE heatmaps for direct protocol comparison. Rows 1-2 show cell radius ($R$) quantification for Optimized SNR 20 and Baseline protocols, respectively. Rows 3-4 show intracellular volume fraction ($V_{in}$) quantification for Optimized SNR 20 and Baseline protocols, respectively. Columns represent SNR levels 5, 10, 20, and 40. Color intensity indicates normalized RMSE (% of parameter maximum). Comparison between row pairs reveals systematic RMSE reduction with optimized protocols across the parameter space.

**Figure S5: Optimized protocols improve T-cell infiltration classification across noise conditions.**

Random Forest classification accuracy at SNR levels 5, 10, 20, and 40 for four protocols: *Optimized SNR 20* (blue), *Optimized SNR 40* (orange), *Baseline2* (green), and *Baseline* (grey). Signals generated from weighted mixtures of two cell populations (tumor cells $R=10 \mu m$; T-cells $R=7 \mu m$) sharing identical diffusion parameters ($V_{in}=0.7, D_{in}=1.1 \mu m^{2}/ms, D_{ex}=0.7 \mu m^{2}/ms, \beta_{ex}=3.0 \mu m^{2}$) at infiltration levels 0%, 15%, and 30%. Optimized protocols achieve higher accuracy across all noise conditions. PCA of signal vectors with varying infiltration levels illustrated enhanced class separability. The *Optimized SNR 20* protocol produced substantially improved separation between classes compared with the Baseline protocol, yielding distinct and well-separated clusters in the dominant principal component space.

**Figure S6. In vivo comparison of baseline and optimized protocols for IMPULSED parameter estimation in canine tumor.**

Parameter maps of cell radius ($R$), intracellular volume fraction ($V_{in}$), and extracellular diffusivity ($D_{ex}$) estimated from the same canine patient undergoing radiation therapy as shown in Figure 4. The acquisition was performed after the first treatment fraction, with one-month interval between the previous acquisition. **(A-C)** *Baseline* protocol results overlaid on b=0 $s/mm^{2}$ reference image. **(D)** Full field-of-view b=0 s/mm² image with red box indicating the region of interest. **(E-G)** *Optimized SNR 20* protocol results overlaid on b=0 $s/mm^{2}$ reference image. **(H)** Full field-of-view b=2000 $s/mm^{2}$ image with red box indicating the region of interest. **(I-K)** Histograms comparing parameter distributions between *Baseline* (blue) and *Optimized* (red) protocols for the entire tumor volume (excluding extreme pixels that reached fitting boundaries). Dashed lines indicate mean values. All parameter maps were estimated using nonlinear least-squares fitting with identical boundary constraints ($R: 0.2-15 \mu m, V_{in}: 0-1, D_{ex}: 0-3.0 \mu m^{2}/ms$). The optimized protocol yields visibly smoother parameter maps with reduced pixel-to-pixel variability and fewer extreme values, demonstrating improved estimation stability compared to the baseline protocol.

**Figure S7. In vivo visualization of IMPULSED parameter evolution after one treatment fraction in canine tumor using the optimized protocol.**

Histograms demonstrate parameter distribution shift before and after radiation treatment with one month interval in the same canine patient. All histograms display data from the entire tumor volume, excluding extreme pixels that reached fitting boundary limits. Dashed lines indicate the mean value for cell radius $R$**,** intracellular volume fraction $V_{in}$, and extracellular diffusion coefficient $D_{ex}$ distribution obtained using the *Optimized SNR 20* protocol.

| Infiltration level Classification Accuracy (%) | | | | |
| --- | --- | --- | --- | --- |
| Protocol | SNR=5 | SNR=10 | SNR=20 | SNR=40 |
| Optimized SNR 20 | 38.13 ± 3.72 | 43.60 ± 2.83 | 55.00 ± 4.68 | **78.47 ± 3.04** |
| Optimized SNR 40 | 37.20 ± 4.60 | 42.33 ± 2.59 | 54.80 ± 3.17 | 75.00 ± 3.19 |
| Baseline | 34.87 ± 3.77 | 37.60 ± 2.80 | 48.47 ± 3.01 | 63.67 ± 3.09 |
| Baseline2 | 33.93 ± 2.41 | 39.13 ± 4.04 | 49.27 ± 2.74 | 65.47 ± 3.36 |

**Table S1: Random Forest classification accuracy for T-cell infiltration detection.**

Classification accuracy for distinguishing T-cell infiltration levels (0%, 15%, 30%) using Random Forest classifier with 10-fold cross-validation. Signals generated from weighted mixtures of tumor cells ($R=10 \mu m$) and T-cells ($R=7 \mu m$) with identical diffusion parameters ($V_{in}=0.7, D_{in}=1.1 \mu m^{2}/ms, D_{ex}=0.7 \mu m^{2}/ms, \beta_{ex}=3.0 \mu m^{2}$). Accuracy values represent mean performance across cross-validation folds. Higher values indicate better discrimination capability.

| EIG rank percentile | Rank (of 800) | EIG | CRLB score |
| --- | --- | --- | --- |
| #1 | 1 | 3.388 | −3.172 |
| 2% | 16 | 3.376 | −3.189 |
| 5% | 40 | 3.369 | −2.531 |
| 10% | 80 | 3.359 | −2.588 |
| 15% | 120 | 3.350 | −2.345 |
| 20% | 160 | 3.338 | −2.488 |
| 30% | 240 | 3.305 | −2.697 |
| 50% | 400 | 3.168 | −1.198 |

**Table S2: EIG-ranked protocols re-scored under the CRLB objective**

Eight representative protocols were selected from the 800 protocols visited by the EIG-based Bayesian-optimization (UR-UVin, SNR=20), corresponding to EIG-rank percentiles #1, 2 %, 5 %, 10 %, 15 %, 20 %, 30 %, and 50 %. Each protocol was re-scored under the CRLB score. Note that higher = better for both EIG and CRLB score.

| Protocol Type | Sequence | $\delta/\Delta(ms)$ | $t_{\mathrm{diff}} (\mathrm{ms})$ | Nonzero $b$-value $(s/mm^{2})$ |
| --- | --- | --- | --- | --- |
| CRLB | PGSE | 10/82 | 78.67 | 424(1), 957(1), 2000(1) |
|  | OGSEn1 | 41/51 | 10.25 | 1(1), 217(1), 290(1), 355(1), 383(1), 402(1), 1220(2) |
|  | OGSEn2 | 31/56 | 3.875 | 42(1), 123(1) |

**Table S3: EIG-ranked protocols re-scored under the CRLB objective**

The CRLB protocol were obtained via Bayesian optimization maximizing CRLB score under UR-UVin prior distribution with SNR=20

| (a) classification performance comparison | | |
| --- | --- | --- |
| Protocol | R accuracy (%) | Vin accuracy(%) |
| Baseline | 74.03 ± 2.53 | 62.60 ± 2.40 |
| Optimized SNR 20 | 91.90 ± 1.82 | 79.40 ± 2.94 |
| CRLB | 74.10 ± 1.55 | 53.73 ± 1.62 |

| (b) Quantification performance comparison | | | |
| --- | --- | --- | --- |
| Protocol | Normalized RMSE of R | Normalized RMSE of Vin | Normalized RMSE of Dex |
| Baseline | 20.13 | 16.01 | 15.56 |
| Optimized SNR 20 | 17.68 | 14.82 | 13.22 |
| CRLB | 20.04 | 15.58 | 16.14 |

**Table S4: Classification and quantification performance comparison of CRLB score based optimization with Baseline and Optimized SNR 20**

(a) Random-forest $R$ and $V_{in}$ classification accuracy (mean ± standard deviation over 10-fold cross-validation). (b)RMSE values normalized by parameter upper bounds ($R: 15 \mu m, D_{in}: 3 \mu m^{2}/ms, V_{in}: 1.0, D_{ex}: 3 \mu m^{2}/ms, \beta_{ex}: 10$). Quantification are all based on the 3-parameter LSQ fit for R, $V_{in}$, and $D_{ex}$, with $D_{in} = 1.58 \mu m^{2}/ms$ and $\beta_{ex} = 2 \mu m^{2}$ fixed.
